# MicroRNA‐188 regulates aging‐associated metabolic phenotype

**DOI:** 10.1111/acel.13077

**Published:** 2019-11-25

**Authors:** Yan Huang, Ye Xiao, Ya Liu, Min Guo, Qi Guo, Fangliang Zhou, Ting Liu, Tian Su, Yuzhong Xiao, Xiang‐Hang Luo

**Affiliations:** ^1^ Department of Endocrinology, Endocrinology Research Center Xiangya Hospital of Central South University Changsha China; ^2^ Department of Biochemistry and Molecular Biology Hunan University of Chinese Medicine Changsha China; ^3^ Department of Endocrinology Changsha Central Hospital Changsha China

**Keywords:** aging‐associated metabolic phenotype, brown adipose tissue, energy expenditure, inguinal white adipose tissue, MicroRNA‐188, PRDM16

## Abstract

With the increasing aging population, aging‐associated diseases are becoming epidemic worldwide, including aging‐associated metabolic dysfunction. However, the underlying mechanisms are poorly understood. In the present study, we aimed to investigate the role of microRNA miR‐188 in the aging‐associated metabolic phenotype. The results showed that the expression of miR‐188 increased gradually in brown adipose tissue (BAT) and inguinal white adipose tissue (iWAT) of mice during aging. MiR‐188 knockout mice were resistant to the aging‐associated metabolic phenotype and had higher energy expenditure. Meanwhile, adipose tissue‐specific miR‐188 transgenic mice displayed the opposite phenotype. Mechanistically, we identified the thermogenic‐related gene *Prdm16* (encoding PR domain containing 16) as the direct target of miR‐188. Notably, inhibition of miR‐188 expression in BAT and iWAT of aged mice by tail vein injection of antagomiR‐188 ameliorated aging‐associated metabolic dysfunction significantly. Taken together, our findings suggested that miR‐188 plays an important role in the regulation of the aging‐associated metabolic phenotype, and targeting miR‐188 could be an effective strategy to prevent aging‐associated metabolic dysfunction.

## Graphical abstract

1) MiR‐188 expression increased gradually in the BAT and iWAT of mice during aging. 2) MiR‐188 knockout mice were resistant to aging‐associated metabolic phenotype and had higher energy expenditure, whereas adipose tissue‐specific miR‐188 transgenic mice had the opposite effects. 3) Inhibiting miR‐188 expression in BAT and iWAT of aged mice by tail vein injection of antagomiR‐188 significantly ameliorated the aging‐associated metabolic phenotype, and targeting miR‐188 might be an effective way to prevent aging‐associated metabolic dysfunction.

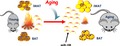

## 1 INTRODUCTION

Aging is often accompanied by an irreversible decline in physiological function, especially metabolic function. The age‐associated metabolic phenotype, including decreased energy expenditure, increased fat mass accumulation, and insulin sensitivity deterioration (Guillory et al., 2017), can ultimately lead to age‐associated metabolic dysfunction, which correlates closely with several disease, such as type 2 diabetes (Lin et al., 2011), fatty liver (Gong, Tas, Yakar, & Muzumdar, 2017; Sheedfar et al., 2014), cardiovascular diseases (Dou et al., 2017), neurodegenerative diseases (Martocchia et al., 2016), and cancer (Topuz et al., 2016). Thus, exploring the underlying mechanisms of the age‐associated metabolic phenotype and developing drugs to treat aging‐associated metabolic dysfunction are of paramount importance.

MicroRNAs (miRNAs) belong to a class of short noncoding regulatory RNAs (22–24 nucleotides) (Li et al., 2015; Treiber, Treiber, & Meister, 2019; Wang et al., 2015; Su et al., 2019), which exert their functions by binding to the 3' untranslated region (3' UTR) or protein coding sequence of target mRNAs (Frankel et al., 2018; Thomou et al., 2017). MiRNAs play crucial roles in regulating many metabolic diseases (Hanin et al., 2018; Langlet et al., 2018; Pankratz et al., 2018; Yu et al., 2018; Zhang et al., 2018). However, the role of miRNAs in the regulation of the aging‐associated metabolic phenotype is unknown. In our previous study, we showed that miR‐188 plays crucial roles in regulating the aging‐associated switch between osteoblast and adipocyte differentiation of bone marrow mesenchymal stem cells (Li et al., 2015). However, a role for miR‐188 in the regulation of the aging‐associated metabolic phenotype remains to be investigated.

Therefore, in the present study, we aimed to investigate the role of miR‐188 in the aging‐associated metabolic phenotype. The results demonstrated that the expression of miR‐188 increased gradually in mouse brown adipose tissue (BAT) and inguinal white adipose tissue (iWAT) during aging. MiR‐188 knockout mice were resistant to the aging‐associated metabolic phenotype and had higher energy expenditure. Adipose tissue‐specific miR‐188 transgenic (Tg) mice had the opposite phenotype. Notably, antagomiR‐188‐mediated inhibition of miR‐188 expression in BAT and iWAT of aged mice ameliorated the aging‐associated metabolic phenotype significantly. The results revealed that miR‐188 regulates the aging‐associated metabolic phenotype and could be a therapeutic target to treat aging‐associated metabolic dysfunction.

## 2 RESULTS

### 2.1 The expression of miR‐188 increased gradually in mouse BAT and iWAT during aging

**Figure 1:**
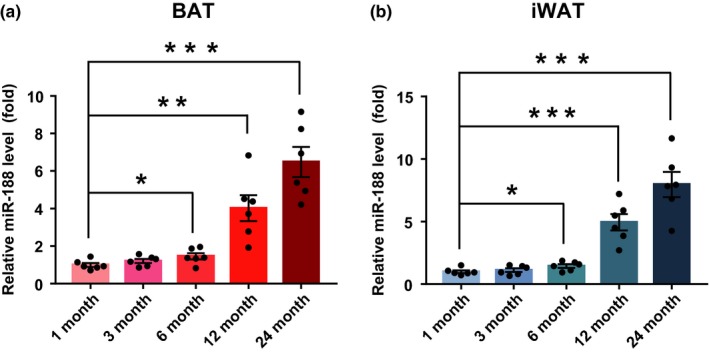
The expression of miR‐188 was gradually increased in BAT and iWAT of mice during aging. (a) The expression of miR‐188 in BAT of WT mice at different ages as indicated. (b) The expression of miR‐188 in iWAT of WT mice at different ages as indicated. Data are shown as means ± *SEM* (*n* = 6). Statistical significance was calculated by one‐way ANOVA followed by Bonferroni posttest, *p < .05; **p < .01; ***p < .001

To determine the potential role of miR‐188 in regulating the aging‐associated metabolic phenotype, first we examined the expression of miR‐188 in the BAT and iWAT of mice at different ages using quantitative real‐time PCR (qPCR) analysis. The results showed that with increasing age, miR‐188 expression increased gradually in the mouse BAT and iWAT (Figure 1a,b), which suggested that miR‐188 plays a role in the regulation of the aging‐associated metabolic phenotype.

### 2.2 MiR‐188 knockout mice were resistant to the aging‐associated metabolic phenotype and had higher energy expenditure

**Figure 2:**
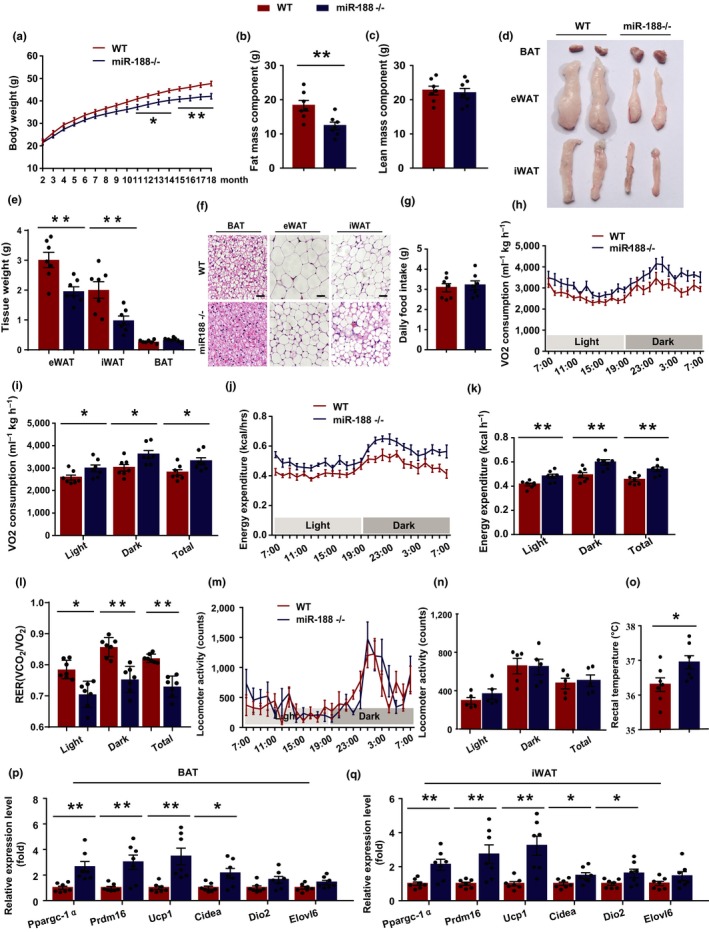
MiR‐188 knockout mice were resistant to the aging‐associated metabolic phenotype and had higher energy expenditure. (a) The body weight curve of male miR‐188 knockout mice and littermate WT mice. (b, c) The body fat mass component and lean mass component of male miR‐188 knockout mice and littermate WT mice at 18‐month old. (d–f) The 18‐month‐old male miR‐188 knockout mice and littermate WT mice were sacrificed and the BAT, eWAT and iWAT were isolated, (d) the gross morphology of BAT, eWAT and iWAT; (e) the weight of BAT, eWAT and iWAT; (f) hematoxylin and eosin staining of BAT, eWAT and iWAT, scale bar: 100 μm. (g) The daily food intake of 18‐month‐old male miR‐188 knockout mice and littermate WT mice. (h–n) The oxygen consumption, energy expenditure, respiratory exchange rate and locomoter activity of 18‐month‐old male miR‐188 knockout mice and littermate WT mice. (o) The rectal temperature of 18‐month‐old male miR‐188 knockout mice and littermate WT mice. (p, q) The mRNA levels of a panel of thermogenic program genes in BAT and iWAT of 18‐month old male miR‐188 knockout mice and littermate WT mice. All the data are shown as means ± *SEM* (*n* = 5–7). Statistical significance was calculated by Student's *t* test or two‐way ANOVA, *p < .05; **p < .01

To further investigate the potential role of miR‐188 in regulating the aging‐associated metabolic phenotype, we generated miR‐188 null mice (Figure S1a). There was no significant difference in body weight between the miR‐188 null mice and their wild‐type (WT) littermates during the first 10 months after birth (Figure 2a). However, with increasing age, the body weights of the miR‐188 null mice became gradually lower than those of their WT littermates (Figure 2a). Quantitative nuclear magnetic resonance (NMR) analyses revealed that the lower body weight of the aged miR‐188 null mice was mainly caused by a decreased fat mass component, while there was no significant difference in the lean mass component between the two groups of mice (Figure 2b,c). Consistently, the gross morphology showed that the size of epididymal white adipose tissue (eWAT) and iWAT were smaller in the aged miR‐188 null mice than in their WT littermates (Figure 2d). In addition, the eWAT and iWAT mass also decreased in aged miR‐188 null mice (Figure 2e). Furthermore, histological staining showed that the adipocytes were smaller in the eWAT and iWAT of the aged miR‐188 null mice (Figure 2f), and the number of lipid droplets in the BAT of the aged miR‐188 null mice also decreased compared with those in their WT littermates (Figure 2f). Taken together, these results suggested that the miR‐188 null mice were resistant to the aging‐associated metabolic phenotype.

Energy homeostasis is maintained by the balance between food intake and energy expenditure (Kim, Seeley, & Sandoval, 2018; Xiao et al., 2017). Our findings showed that the aged miR‐188 null mice had an unchanged food intake (Figure 2g); however, their oxygen (O_2_) consumption and energy expenditure (EE) were significantly higher compared with those of their WT littermates (Figure 2h–i), which suggested that the decreased body fat mass of the aged miR‐188 null mice was largely the result of increased energy expenditure. Under a normal environmental temperature, energy expenditure mainly comprises thermogenesis and physiological activities (Cui et al., 2016). Although their physiological activities were not changed (Figure 2m,n), the aged miR‐188 null mice had a significantly higher body temperature than their WT littermates (Figure 2o). The higher body temperature of aged miR‐188 null mice was likely caused by increased thermogenesis, because the mRNA expression levels of a subset of thermogenesis‐related genes, such as *Ppargc‐1α* (encoding peroxisome proliferator‐activated receptor gamma coactivator 1‐alpha), *Prdm16* (encoding PR domain containing 16), *Ucp1* (encoding Uncoupling protein 1), and *Cidea* (encoding cell death‐inducing DFF‐like effector A) were increased significantly in the BAT and iWAT of the aged miR‐188 null mice (Figure 2p,q).

Thus, these findings suggested that the decreased body fat mass of the aged miR‐188 null mice was largely caused by increased energy expenditure.

### 2.3 Adipose tissue‐specific miR‐188 Tg mice were prone to develop the aging‐associated metabolic phenotype and had lower energy expenditure

**Figure 3:**
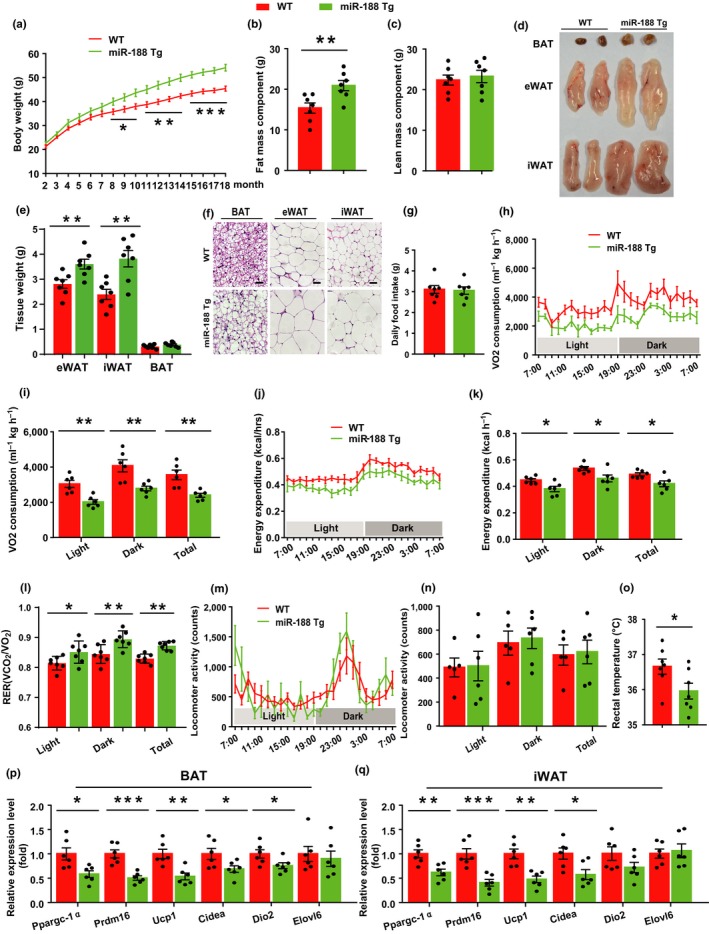
Adipose tissue‐specific miR‐188 Tg mice were prone to develop the aging‐associated metabolic phenotype and had lower energy expenditure. (a) The body weight curve of male miR‐188 Tg mice and littermate WT mice. (b, c) The body fat mass component and lean mass component of male miR‐188 Tg mice and littermate WT mice at 18‐month old. (d–f) The 18‐month‐old male miR‐188 Tg mice and littermate WT mice were sacrificed and the BAT, eWAT and iWAT were isolated, (d) the gross morphology of BAT, eWAT, and iWAT; (e) the weight of BAT, eWAT, and iWAT; (f) hematoxylin and eosin staining of BAT, eWAT, and iWAT, scale bar: 100 μm. (g) The daily food intake of male 18‐month old miR‐188 Tg mice and littermate WT mice. (h–n) The oxygen consumption, energy expenditure, respiratory exchange rate and locomoter activity of 18‐month‐old male miR‐188 Tg mice and littermate WT mice. (o) The rectal temperature of 18‐month‐old male miR‐188 Tg mice and littermate WT mice. (p, q) The mRNA levels of a panel of thermogenic program genes in BAT and iWAT of 18‐month old male miR‐188 Tg mice and littermate WT mice. All the data are shown as means ± *SEM* (*n* = 5–7). Statistical significance was calculated by Student's *t* test or two‐way ANOVA, *p < .05; **p < .01; ***p < .001

To further verify the findings reported above, we generated Tg mice that overexpressed miR‐188 in an adipose tissue‐specific manner (Figure S2a). The body weights of the miR‐188 Tg mice showed no significant differences compared with those of their WT littermates during the first 7 months after birth (Figure 3a). However, with increasing age, the body weight of the miR‐188 Tg mice gradually became higher than that of their WT littermates (Figure 3a). The increased body weight of aged miR‐188 Tg mice was mainly caused by an increased fat mass component, while the lean mass component showed no significant differences between the two groups of mice (Figure 3b,c). Consistently, when the aged miR‐188 Tg mice and their WT littermates were sacrificed and the adipose tissues were isolated, the gross size and mass of the eWAT and iWAT were increased in the aged miR‐188 Tg mice (Figure 3d,e). In addition, histological staining of the BAT, eWAT, and iWAT in the two groups of mice revealed that the adipocytes were larger in the eWAT and iWAT of the aged miR‐188 Tg mice compared with those in their WT littermates (Figure 3f). In addition, the size of the lipid droplets in the BAT of the aged miR‐188 Tg mice also increased (Figure 3f). These findings suggested that the adipose tissue‐specific miR‐188 Tg mice were prone to developing the aging‐associated metabolic phenotype.

Furthermore, we investigated the energy metabolism of aged miR‐188 Tg mice and their WT littermates. The daily food intake showed no significant differences (Figure 3g); however, the O_2_ consumption and energy expenditure decreased significantly in the aged miR‐188 Tg mice compared with those in their WT littermate (Figure 3h–l). Although their physiological activities showed no obvious changes (Figure 3m,n), the body temperature of the aged Tg mice was significantly lower than that of their WT littermates (Figure 3o). The lower body temperature observed in the aged miR‐188 Tg mice was most likely caused by decreased thermogenesis, because the mRNA levels of thermogenesis‐related genes, *Ppargc‐1α*, *Prdm16*, *Ucp1*, and *Cidea* were all decreased in the BAT and iWAT of the aged miR‐188 Tg mice compared with those in their WT littermates (Figure 3p,q).

Taken together, these results suggested that the increased body fat mass of the aged adipose tissue‐specific miR‐188 transgenic mice was most likely caused by decreased energy expenditure.

### 2.4 *Prdm16* is a potential target of miR‐188 in the regulation of the aging‐associated metabolic phenotype

**Figure 4:**
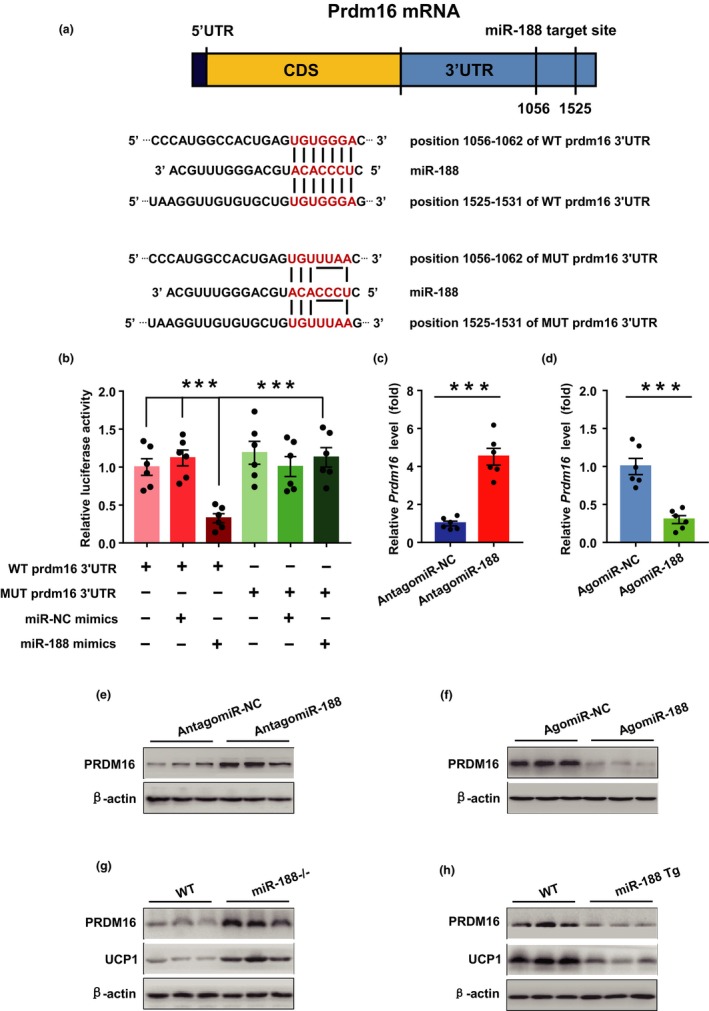
*Prdm16* is a potential target of miR‐188 in the regulation of the aging‐associated metabolic phenotype. (a) Schematic of the sequence that miR‐188 targets the WT or mutated 3' UTR of *Prdm16* mRNA. (b) The luciferase activity of *Prdm16* WT or mutated 3' UTR reporter plasmids co‐transfected with miR‐188 mimics or miR‐NC mimics in HEK293 cells as indicated. (c, d) The mRNA levels of *Prdm16* in primary cultured brown adipocytes transfected with miR‐188 mimics or miR‐NC mimics. (e, f) The protein levels of PRDM16 in primary cultured brown adipocytes transfected with miR‐188 mimics or miR‐NC mimics. (g, h) The protein levels of PRDM16 and UCP1 in BAT of 18‐month old miR‐188 knockout mice and miR‐188 Tg mice as indicated. Data are shown as means ± *SEM* (*n* = 5–7 in g, h), the cell experiments were repeated for at least three times. Statistical significance was calculated by Student's *t* test or two‐way ANOVA, ***p < .001

To further investigate the underlying mechanism by which miR‐188 regulates the aging‐associated metabolic phenotype, we used online tools, including TargetScan 6.2 (http://www.targetscan.org/) and miRanda (http://www.microrna.org/microrna/), to predict the potential targets of miR‐188 (He, Han, et al., 2018a; Savita & Karunagaran, 2013). Among the predicted target genes, our attention was drawn to *Prdm16*, because sequence analysis revealed there was a conserved miR‐188 binding site in its 3' UTR (Figure 4a), meanwhile PRDM16 has been reported to function as a key transcription factor that regulates the expression of thermogenic program genes in brown and beige adipocytes (Cohen et al., 2014; Harms et al., 2014). To explore the association between *Prdm16* and miR‐188, we generated luciferase reporter plasmids containing the wild‐type 3' UTR of *Prdm16* (WT *Prdm16* 3' UTR). HEK293 cells were co‐transfected with the WT *Prdm16* 3' UTR luciferase reporter plasmids and miR‐188 mimics. The results showed that miR‐188 mimic overexpression significantly inhibited the luciferase activity (Figure 4b). However, this inhibition was largely abolished when the binding site of miR‐188 in the 3' UTR of *Prdm16* was mutated (Figure 4b). In addition, in cultured primary brown adipocytes, the mRNA and protein levels of PRDM16 increased when transfected with antagomiR‐188 or decreased when transfected with agomiR‐188 (Figure 4c–f). Consistently, in the BAT of aged miR‐188 knockout mice the protein levels of PRDM16 and UCP1 also increased, whereas in the aged miR‐188 Tg mice, the protein levels of PRDM16 and UCP1 decreased (Figure 4g,h). These findings suggested that *Prdm16* is a potential target of miR‐188 in the regulation of the aging‐associated metabolic phenotype.

### 2.5 Administration of antagomiR‐188 to aged mice ameliorated aging‐associated metabolic phenotype and stimulated energy expenditure

**Figure 5:**
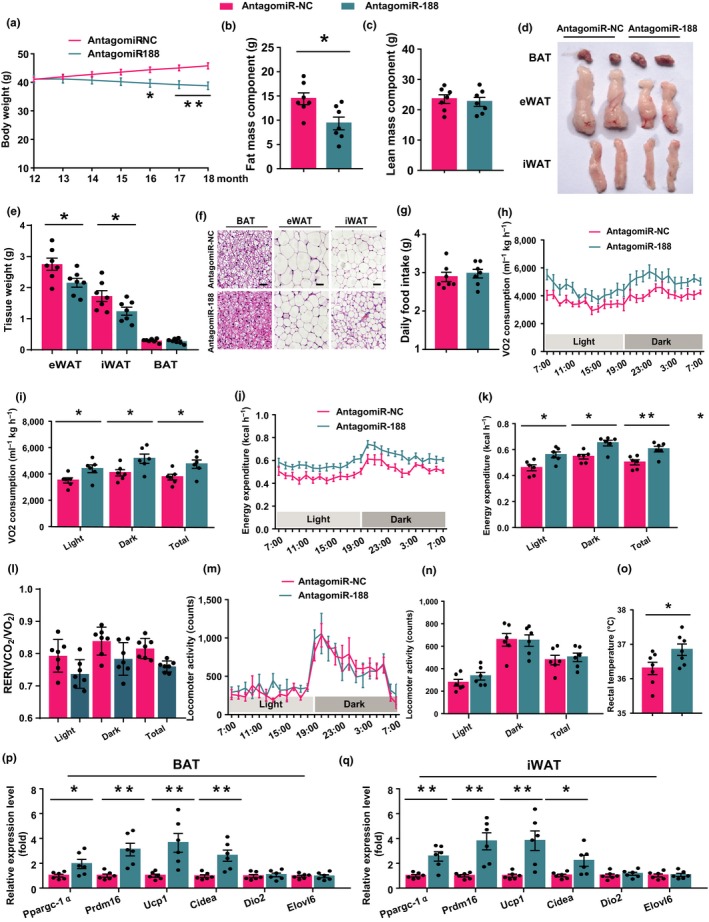
Administration of antagomiR‐188 to aged mice ameliorated aging‐associated metabolic phenotype and stimulated energy expenditure. (a) The body weight curve of aged male mice administrated with antagomiR‐188 or antagomiR‐NC for six months. (b, c) The body fat mass component and lean mass component of aged male mice administrated with antagomiR‐188 or antagomiR‐NC for six months. (d–f) The aged male mice administrated with antagomiR‐188 or antagomiR‐NC for six months were sacrificed and the BAT, eWAT, and iWAT were isolated, (d) the gross morphology of BAT, eWAT, and iWAT; (e) the weight of BAT, eWAT, and iWAT; (f) hematoxylin and eosin staining of BAT, eWAT, and iWAT, scale bar: 100 μm. (g) The daily food intake of aged male mice administrated with antagomiR‐188 or antagomiR‐NC for six months. (h–n) The oxygen consumption, energy expenditure, respiratory exchange rate, and locomoter activity of aged male mice administrated with antagomiR‐188 or antagomiR‐NC for six months. (o) The rectal temperature of aged male mice administrated with antagomiR‐188 or antagomiR‐NC for six months. (p, q) The mRNA levels of a panel of thermogenic program genes in BAT and iWAT of aged male mice administrated with antagomiR‐188 or antagomiR‐NC for six months. All the data are shown as means ± *SEM* (*n* = 6–7). Statistical significance was calculated by Student's *t* test or two‐way ANOVA, *p < .05; **p < .01

The findings above suggested that miR‐188 is a potential therapeutic target for aging‐associated metabolic dysfunction. To inhibit the expression of miR‐188 in the BAT and iWAT of aged mice, we injected antagomiR‐188 to the tail veins of aged mice, as reported previously (Zhang et al., 2016). Six months later, we found that antagomiR‐188 injection significantly inhibited the expression of miR‐188 in the BAT and iWAT of the aged mice (Figure S3a). Monthly body weight measurements of the mice revealed that antagomiR‐188 injection gradually decreased the body weight of the aged mice (Figure 5a). Further studies showed that the decreased body weight of the antagomiR‐188‐injected mice was mainly caused by a decreased fat mass component, because the lean mass component showed no obvious changes between the antagomiR‐188‐injected mice and the antagomiR‐NC controls (Figure 5b,c). Consistently, the gross size and mass of the eWAT and iWAT in the antagomiR‐188‐injected mice also decreased (Figure 5d,e). In addition, histological staining showed that antagomiR‐188 injection decreased the size of the adipocytes in the eWAT and iWAT of the aged mice (Figure 5f). The number of lipid droplets in the BAT of the aged antagomiR‐188‐injected mice also decreased (Figure 5f). Taken together, these results suggested that tail vein injection of antagomiR‐188 ameliorated the aging‐associated metabolic phenotype.

To evaluate the effects of antagomiR‐188 injection on energy metabolism, the food intake and energy expenditure of aged antagomiR‐188‐injected mice and the controls were measured. The food intake was not affected by antagomiR‐188 injection (Figure 5g); however, the O_2_ consumption and energy expenditure increased significantly increased after antagomiR‐188 injection (Figure 5h–l). The increased energy expenditure observed in the aged antagomiR‐188‐ injected mice was most likely caused by increased thermogenesis, because the two groups of mice showed no differences in their physiological activities (Figure 5m,n); however, the body temperature was significantly higher in the antagomiR‐188‐injected mice than in the controls (Figure 5o). Consistently, the mRNA expression of thermogenesis‐related genes, *Ppargc‐1α*, *Prdm16*, *Ucp1*, and *Cidea* were significantly increased in the BAT and iWAT of the antagomiR‐188‐injected mice (Figure 5p,q), and the protein levels of PRDM16 and UCP1 were also increased in the BAT of the antagomiR‐188‐injected mice compared with those in the controls (Figure S6h).

Taken together, these findings suggested that administration of antagomiR‐188 ameliorated the aging‐associated metabolic phenotype by increasing energy expenditure. And targeting miR‐188 might be an effective way to prevent aging‐associated metabolic dysfunction.

## 3 DISCUSSION

MiRNAs have been reported to participate in the regulation of a wide variety of metabolic diseases. However, a role for microRNAs in the regulation of the aging‐associated metabolic phenotype has not been reported. In our previous study, we demonstrated that miR‐188 is an important regulator of aging‐associated bone mass loss (Li et al., 2015). Therefore, we questioned whether miR‐188 also functions in the aging‐associated metabolic phenotype.

The results of the present study showed that the expression of miR‐188 gradually increased in the BAT and iWAT of mice during aging. For the WT mice, some metabolic changes gradually appeared during aging, including body weight gain and fat mass accumulation. However, the aged mice with miR‐188 knockout did not develop these phenotypes, while the adipose tissue‐specific miR‐188 Tg mice developed these phenotypes to a greater extent. These findings revealed that miR‐188 exerts an important role in the regulation of the aging‐associated metabolic phenotype.

Energy homeostasis is maintained by a balance between food intake and energy expenditure (Kim et al., 2018; Xiao et al., 2017). In the aged miR‐188 null mice and aged adipose tissue‐specific miR‐188 transgenic mice, the food intake was unchanged; however, the oxygen consumption and energy expenditure were significantly increased or decreased compared with those in the corresponding control mice, respectively. Under normal environmental conditions, energy expenditure occurs through physiological activities and thermogenesis (Cui et al., 2016; Deng et al., 2017; Wyler, Lord, Lee, Elmquist, & Liu, 2017). The mice's physiological activities were unchanged; however, the expression of thermogenesis‐related genes increased or decreased significantly in the BAT and iWAT of aged miR‐188 null mice or aged adipose tissue‐specific miR‐188 transgenic mice, respectively. Moreover, except for serum triglyceride, there was little difference between the genetic mice and their controls in terms of glucose and lipid metabolism (Figure S3 and Figure S4). Thus, these results suggested that miR‐188 regulates the aging‐associated metabolic phenotype largely by affecting the thermogenesis.

Further analysis identified *Prdm16* as a downstream effector of miR‐188, which may participate in the regulation of aging‐associated metabolic phenotype. Prdm16 is a key transcription factor that regulates the expression of a panel of thermogenic program genes in brown adipocytes and beige adipocytes (Cohen et al., 2014; Harms et al., 2014) and plays an important role in maintaining iBAT and scWAT identity (Seale et al., 2011, 2007). The results of the present study showed that miR‐188 could bind directly to the 3' UTR of *Prdm16* mRNA to inhibit its expression. This suggested that *Prdm1*6 maybe the downstream target of miR‐188 in the regulation of the aging‐associated metabolic phenotype.

To further evaluate the therapeutic potential of targeting miR‐188 in the treatment of aging‐associated metabolic dysfunction, we injected antagomiR‐188 into the aged mice via tail vein injection. Administration of antagomiR‐188 significantly inhibited the expression of miR‐188 in the BAT and iWAT of the aged mice. Furthermore, antagomiR‐188 injection ameliorated aging‐associated metabolic phenotype significantly. Taken together, these findings revealed a role of miR‐188 in the regulation of the aging‐associated metabolic phenotype, suggesting that targeting miR‐188 might be an effective way to prevent aging‐associated metabolic dysfunction.

## 4 EXPERIMENTAL PROCEDURES

### 4.1 Animals

C57BL/6J wild‐type (WT) mice were obtained from Shanghai Laboratory Animals Co. Ltd (Shanghai, China). The miR‐188 null mice were generated by transcription activator‐like effector nuclease (TALEN) technique as reported previously (Li et al., 2015). To generate adipose tissue‐specific miR‐188 transgenic (Tg) mice, first, the pre‐miR‐188 cDNA (synthesized by Shanghai sangon Co.) was subcloned into a plasmid containing Fabp4 (Ap2) promoter (Shi et al., 2014), resulting in Ap2‐pre‐miR‐188 vector. Then, the Ap2‐pre‐miR‐188 plasmid and empty plasmid were transfected into 3T3‐L1 adipocytes by Lipofectamine 2000 (invitrogen), the expression of miR‐188 in 3T3‐L1 adipocytes was measured by qRT‐PCR analysis. After the successful construction of Ap2‐pre‐miR‐188 plasmid, the plasmids were linearized and purified, then micro‐injected into C57BL/6J F2 mouse oocytes, and the injected oocytes were then surgically transferred into pseudopregnant C57BL/6J dams. Two lines with high levels of miR‐188 expression in BAT and iWAT were selected from six transgenic founders and crossed with C57BL/6J strain for six generations to obtain offsprings with a defined genetic background. One line with a fivefold overexpression of miR‐188 was extensively studied. The WT mice were used as controls. All mice were kept in C57BL/6J background and maintained in standard, specific pathogen‐free facility of the Laboratory Animal Research Center at Central South University, with a 12‐hr dark/light cycle and 4–5 mice per cage. In this study, all mice were kept on a standard normal chow diet purchased from Shanghai Laboratory Animals Co. Ltd (Shanghai, China). All animal care protocols and experiments were reviewed and approved by the Animal Care and Use Committees of the Laboratory Animal Research Center at Xiangya Medical School of Central South University, and this study was compliant with all relevant ethical regulations regarding animal research.

### 4.2 Intravenous administration of miR‐188 antagomir

The miRNA antagomir is a chemically modified, cholesterol conjugated, single‐stranded RNA analog that complements the miRNAs. It efficiently and specifically silences endogenous miRNAs. AntagomiR‐188 and its negative control (NC) were synthesized by RiboBio Co. For tail vein injection of antagomiR‐NC or antagomiR‐188, the aged mice were received antagomiR‐188 once a week (10 mg/kg body weight, 0.2 ml for each injection) for six months before conducting metabolic parameters measurements. The antagomir negative control was administered at the same dose and injection intervals. The functional inhibition by the administered antagomirs in vivo was verified by qRT‐PCR.

### 4.3 Metabolic parameter measurements

The fat mass component and lean mass component of mice were measured by a nuclear magnetic resonance (NMR) system (Bruker, Rheinstetten, Germany). Indirect calorimetry was conducted in a comprehensive laboratory animal‐monitoring system (Columbus Instruments, Columbus, OH), as described previously (Xiao et al., 2017, 2016). Rectal temperature of mice was measured at 14:00 pm by a rectal probe attached to a digital thermometer (Physitemp, NJ, USA). The measurement of daily food intake was also conducted as reported previously (Xiao et al., 2017, 2016).

### 4.4 Histological analysis of tissues

Paraformaldehyde‐fixed, paraffin‐embedded sections of BAT, eWAT, and iWAT were stained with hematoxylin and eosin (H&E) for histology.

### 4.5 Luciferase activity assays

The luciferase activity assays were conducted as reported previously (Li et al., 2009; Yang et al., 2017). Generally, the wild‐type (WT) PRDM16 3'UTR firefly luciferase reporter plasmids or PRDM16 3'UTR firefly luciferase reporter plasmids with the potential miR‐188 binding site mutated were co‐transfected with miR‐188 mimics or miR‐NC mimics to the HEK293 cells, respectively. Renilla luciferase reporter plasmids were also transfected at the same time as internal control. 48 hr posttransfections, firefly and renilla luciferase activities were measured by a Dual‐Glo Luciferase Assay System (Promega).

### 4.6 Primary culture of brown adipocytes

The primary culture of brown adipocytes was performed as described previously (He, Tang, et al., 2018b; Hu et al., 2015). Briefly, the brown adipose tissue from three weeks old C57/BL6J mice was isolated and minced quickly. Then, the tissue pieces were digested in an isolation buffer containing 123 mM NaCl, 5mM KCl, 1.3 mM CaCl2, 5 mM glucose, 100 mM HEPES, 4% BSA and 1.5 mg/ml collagenase B (Roche) for 45 min at 37°C. After filtered, centrifuged and washed with PBS, the preadipocytes were cultured in an adipocyte culture medium (DMEM plus GlutaMAX, penicillin and streptomycin, and 10% FBS). The preadipocytes were grown to confluence before adipocyte differentiation, which was induced by a differentiation buffer containing 5 mM dexamethasone, 0.02 mM insulin, 0.5 mM isobutylmethylxanthine, 1 nM T3, 125 mM indomethacin and 1 mM rosiglitazone. Two days after the induction, cells were incubated in fresh adipocyte culture medium containing 0.02 mM insulin, 1 nM T3 and 1 mM rosiglitazone. The culture medium was changed every other days until the appearance of multiple small lipid droplets in the cytoplasma.

### 4.7 Western blot analysis

The Western Blot analysis was conducted as previously described (Li et al., 2015, 2009), primary antibodies: anti‐UCP1 was purchased from Cell Signalling Technology (#14670), anti‐PRDM16 was purchased from Abcam (#ab202344), anti‐β‐actin was purchased from Proteintech (#HRP‐60008). All validation information could be found on the manufacturer's website.

### 4.8 RNA isolation and quantitative real‐time PCR (qPCR)

The RNA isolation and qPCR analysis were performed as described previously (Li et al., 2015, 2009). The primer pairs used in this study are listed in Table S1.

### 4.9 Quantification and statistical analysis

All the results are expressed as means ± S.E.M. Each data point derived from qRT‐PCR analysis represents an average of at least three technical replicates. The statistical significance of the differences between various treatments or groups was measured by either Student's *t* test or ANOVA followed by Bonferroni posttest. Data analyses were performed using GraphPad Prism 7.0. *p* < .05 was considered statistically significant, *p < .05; **p < .01; ***p < .001.

## Competing Financial Interests

The authors declare no competing financial interests.

## AUTHORS’ CONTRIBUTION

YZ.X and XH.L designed the experiments and wrote the manuscript; Y.H, Y.L and YZ.X performed most of the experiments; Y.X, Q.G, FL.Z, T.L and T.S helped to collect samples. YZ.X and XH.L is the guarantor of this work and, as such, has full access to all the data in this study and takes responsibility for the integrity of the data and the accuracy of the data analysis.

## Data Availability

The data that support the findings of this study are available from the corresponding author upon reasonable request.
