# Supplementary figures and images for: MicroRNA‐188 regulates aging‐associated metabolic phenotype
Source: Aging Cell. 2019 Nov 25;19(1):e13077. doi: 10.1111/acel.13077 (PMC6974730; doi:10.1111/acel.13077)

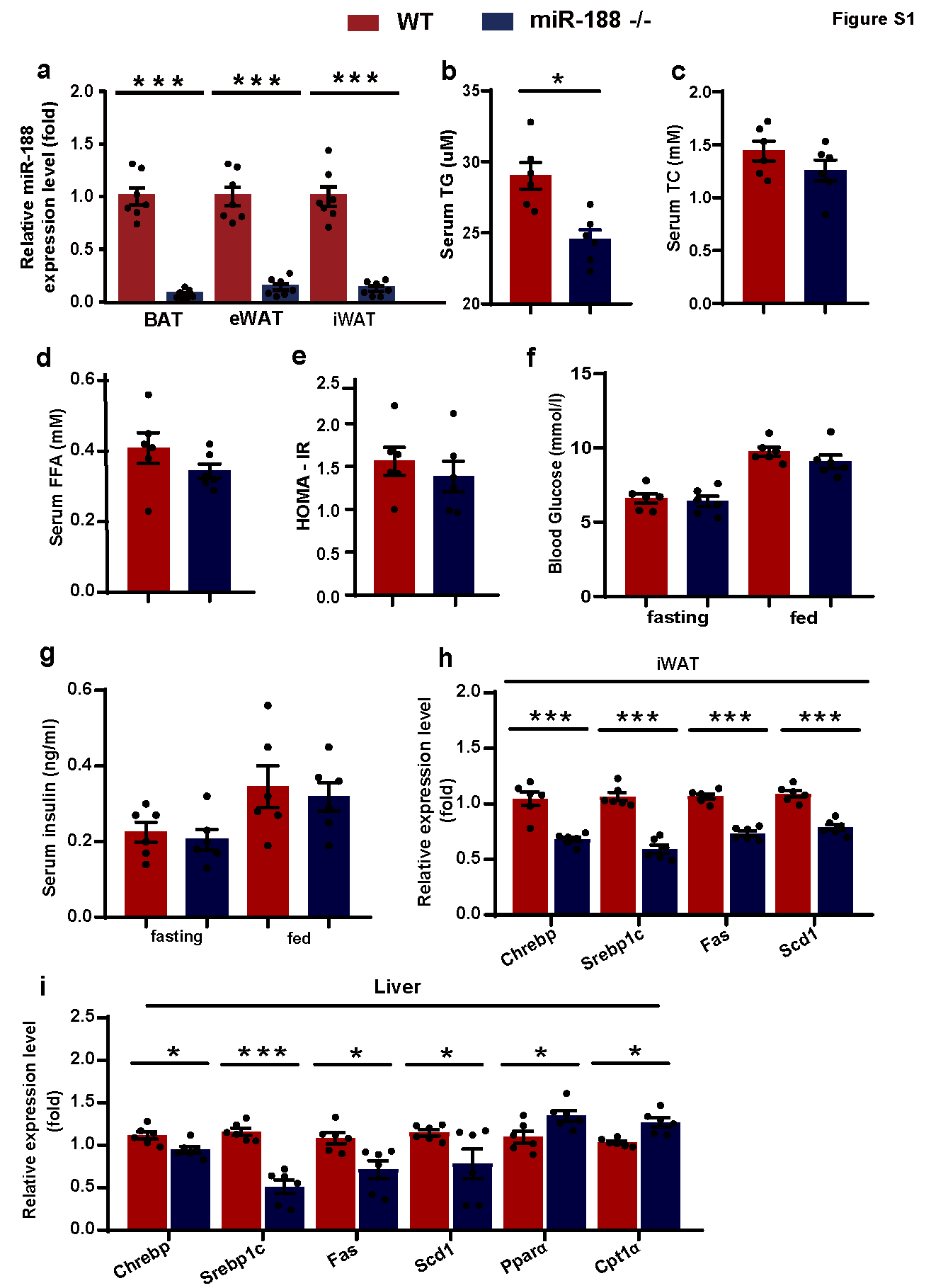

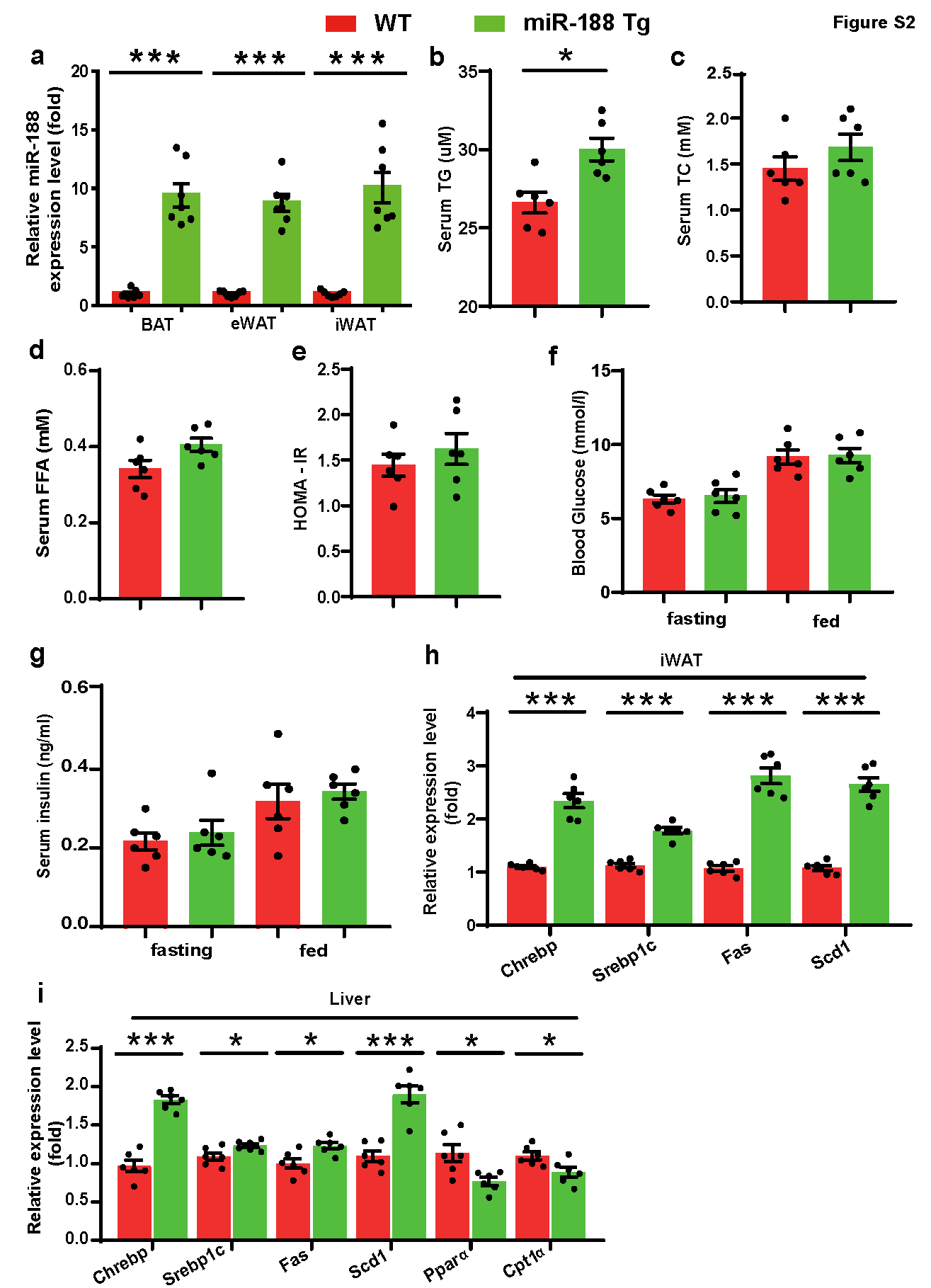

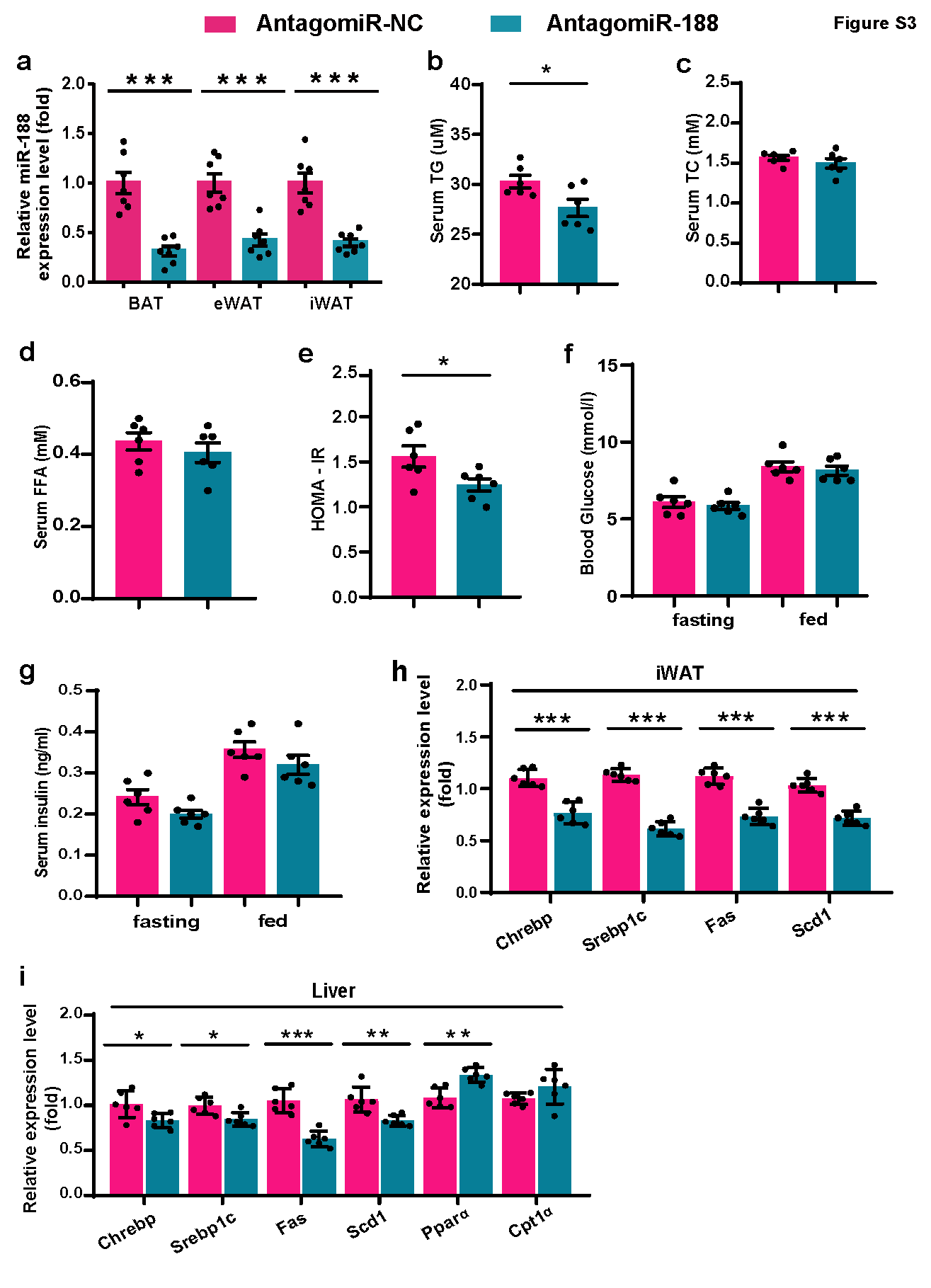

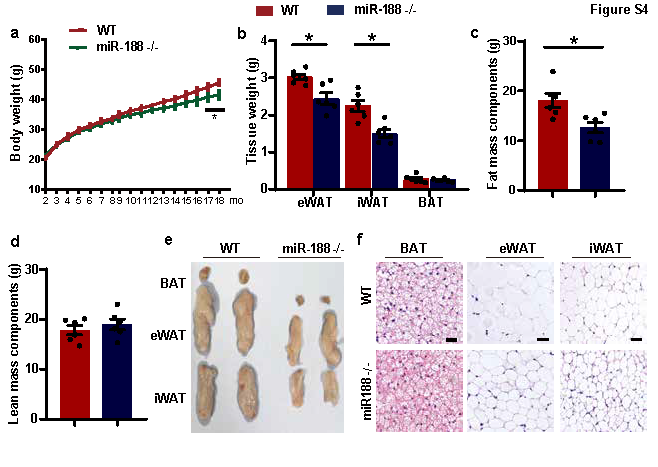

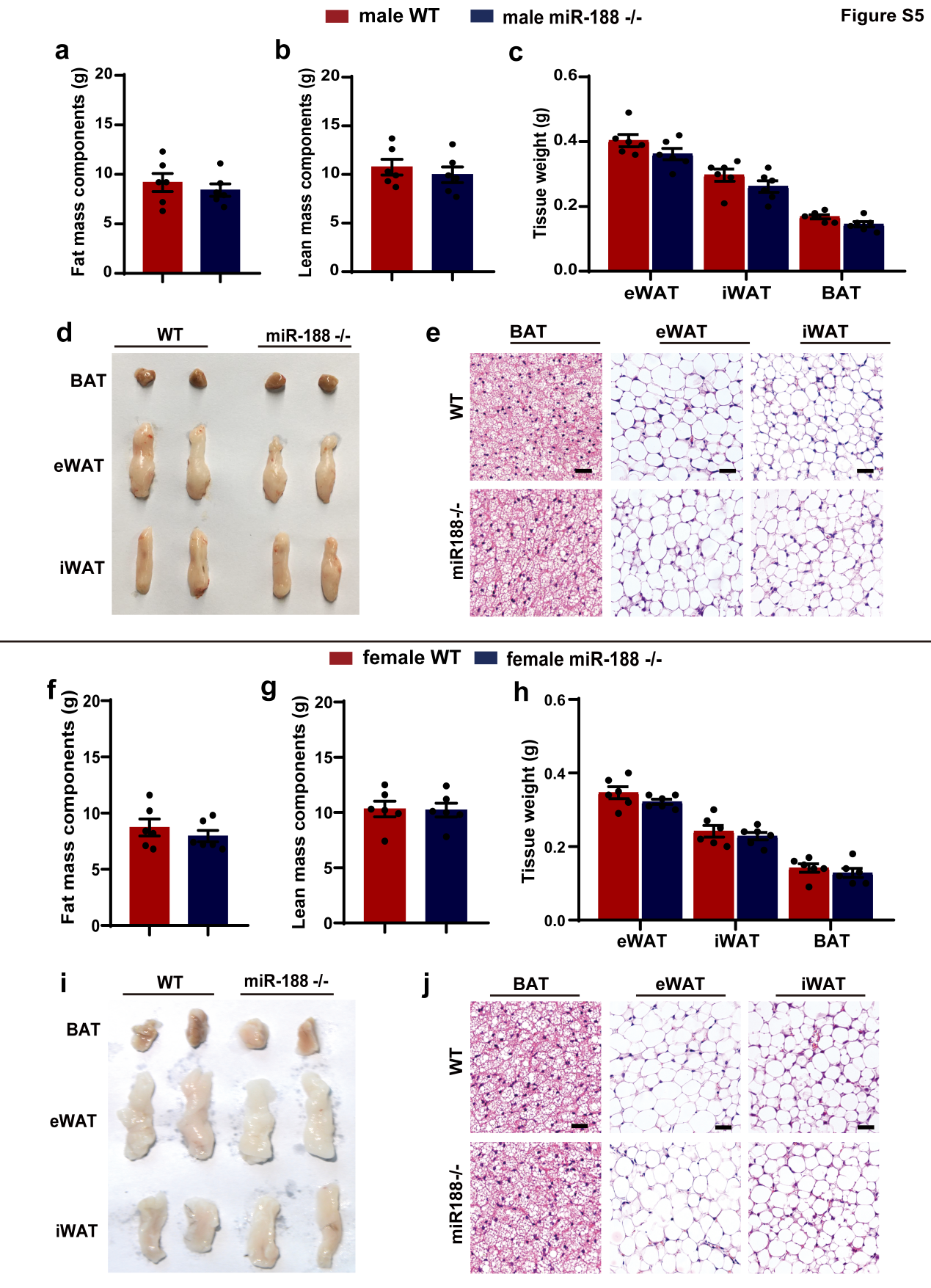

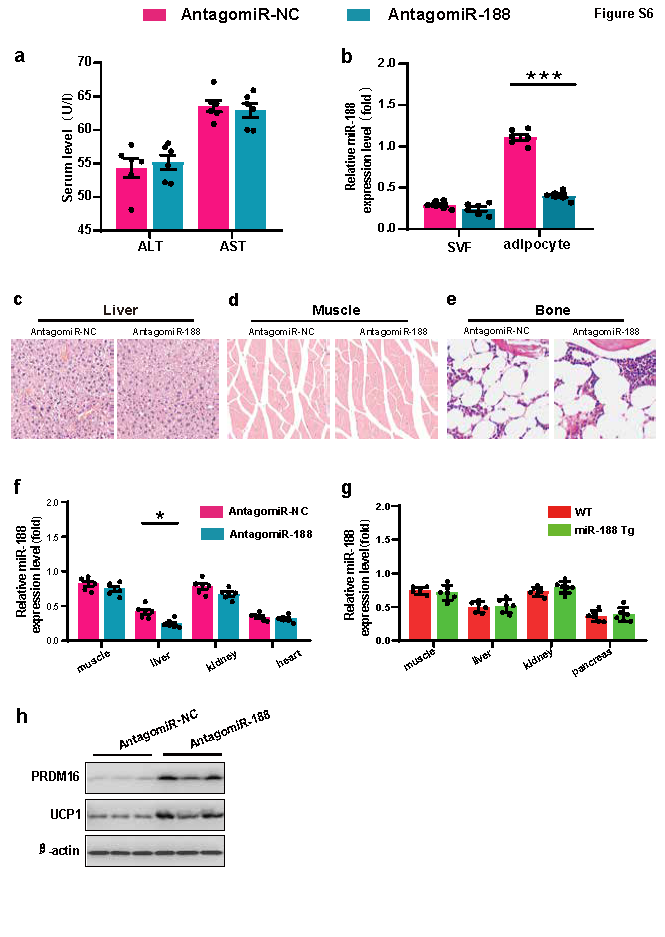

Supplement: Supplementary file 1 [file ACEL-19-e13077-s001.docx]
