# Supplementary material for: MicroRNA‐188 regulates aging‐associated metabolic phenotype
Source: Aging Cell. 2019 Nov 25;19(1):e13077. doi: 10.1111/acel.13077 (PMC6974730; doi:10.1111/acel.13077)
Supplement: Supplementary file 2 [file ACEL-19-e13077-s002.docx]

| **Target Gene** | **Sense Primer** | **Antisense Primer** |
| --- | --- | --- |
| Ppargc-1α | 5'-CCCTGCCATTGTTAAGACC-3' | 5'-TGCTGCTGTTCCTGTTTTC-3' |
| Prdm16 | 5'-CCACCAGCGAGGACTTCAC-3' | 5'-GGAGGACTCTCGTAGCTCGAA-3' |
| Ucp1 | 5'-CTGCCAGGACAGTACCCAAG-3' | 5'-TCAGCTGTTCAAAGCACACA-3' |
| Cidea | 5'-ATCACAACTGGCCTGGTTACG-3' | 5'-TACTACCCGGTGTCCATTTCT-3' |
| Dio2 | 5'-ATGGGACTCCTCAGCGTAGAC-3' | 5'-ACTCTCCGCGAGTGGACTT-3' |
| Elovl6 | 5'-TGCCATGTTCATCACCTTGT-3' | 5'-TGCTGCATCCAGTTGAAGAC-3' |
| Chrebp | 5'-CCTCACTTCACTGTGCCTCA-3' | 5'-ACAGGGGTTGTTGTCTCTGG-3' |
| Srebp1c | 5'-GGAGCCATGGATTGCACATT-3' | 5'-GGAAGTCACTGTCTTGGTTGTTGA-3' |
| Fas | 5'-AAGTTGCCCGAGTCAGAGAA-3' | 5'-CGTCGAACTTGGAGAGATCC-3' |
| Scd1 | 5'-GCGATACACTCTGGTGCTCA-3' | 5'-CCCAGGGAAACCAGGATATT-3' |
| Cpt1α | 5'-ATCGTGGTGGTGGGTGTGATAT-3' | 5'-ACGCCACTCACGATGTTCTTC-3' |
| Pparα | 5'-AGAGCCCCATCTGTCCTCTC-3' | 5'-ACTGGTAGTCTGCAAAACCAAA-3' |

**Table S1. List of oligonucleotide primer pairs used in RT-PCR analysis**
